# Transcript mapping of *Cotton leaf curl Burewala virus* and its cognate betasatellite, Cotton leaf curl Multan betasatellite

**DOI:** 10.1186/1743-422X-9-249

**Published:** 2012-10-29

**Authors:** Fazal Akbar, Rob W Briddon, Franck Vazquez, Muhammad Saeed

**Affiliations:** 1 Agricultural Biotechnology Division, National Institute for Biotechnology and Genetic Engineering, Jhang Road, Faisalabad, Pakistan; 2 Zürich-Basel Plant Science Center, Part of the Swiss Plant Science Web, Botanical Institute of the University of Basel, Schönbeinstraße 6 CH-4056, Basel, Switzerland

**Keywords:** Begomovirus, Cotton leaf curl disease, *Cotton leaf curl Burewala virus*, Betasatellite, Cotton leaf curl Multan betasatellite, Transcription

## Abstract

**Background:**

Whitefly-transmitted geminiviruses (family *Geminiviridae*, genus *Begomovirus*) are major limiting factors for the production of numerous dicotyledonous crops throughout the warmer regions of the world. In the Old World a small number of begomoviruses have genomes consisting of two components whereas the majority have single-component genomes. Most of the monopartite begomoviruses associate with satellite DNA molecules, the most important of which are the betasatellites. Cotton leaf curl disease (CLCuD) is one of the major problems for cotton production on the Indian sub-continent. Across Pakistan, CLCuD is currently associated with a single begomovirus (*Cotton leaf curl Burewala virus* [CLCuBuV]) and the cotton-specific betasatellite Cotton leaf curl Multan betasatellite (CLCuMuB), both of which have recombinant origins. Surprisingly, CLCuBuV lacks *C2*, one of the genes present in all previously characterized begomoviruses. Virus-specific transcripts have only been mapped for few begomoviruses, including one monopartite begomovirus that does not associate with betasatellites. Similarly, the transcripts of only two betasatellites have been mapped so far. The study described has investigated whether the recombination/mutation events involved in the evolution of CLCuBuV and its associated CLCuMuB have affected their transcription strategies.

**Results:**

The major transcripts of CLCuBuV and its associated betasatellite (CLCuMuB) from infected *Nicotiana benthamiana* plants have been determined. Two complementary-sense transcripts of ~1.7 and ~0.7 kb were identified for CLCuBuV. The ~1.7 kb transcript appears similar in position and size to that of several begomoviruses and likely directs the translation of C1 and C4 proteins. Both complementary-sense transcripts can potentially direct the translation of C2 and C3 proteins. A single virion-sense transcript of ~1 kb, suitable for translation of the *V1* and *V2* genes was identified. A predominant complementary-sense transcript was also confirmed for the betasatellite.

**Conclusions:**

Overall, the transcription of CLCuBuV and the recombinant CLCuMuB is equivalent to earlier mapped begomoviruses/betasatellites. The recombination events that featured in the origins of these components had no detectable effects on transcription. The transcripts spanning the mutated C2 gene showed no evidence for involvement of splicing in restoring the ability to express intact C2 protein.

## Background

Viruses of the *Geminiviridae* family have circular single-stranded (ss)DNA genomes and classify into four genera according to their host range, insect vector and genome organization [1]. They are widely distributed throughout the world and infect either monocotyledonous or dicotyledonous hosts. Geminiviruses of the genus *Begomovirus* are transmitted by the whitefly *Bemisia tabaci* and have genomes that consist of either a single or two ssDNA components. The two components of bipartite begomoviruses are known as DNA A and DNA B and both are, for most species, essential for symptomatic infection of plants. Monopartite begomoviruses are often associated with DNA satellites known as alphasatellites and betasatellites [2]. The satellite-associated begomoviruses are widespread in the Old World and represent the largest group of begomoviruses, outnumbering both the truly monopartite and the bipartite begomoviruses.

Geminiviruses replicate *via* a double-stranded (ds) DNA intermediate that is also used as a template for transcription. Transcription is bidirectional to generate mRNAs diverging from an intergenic region. Transcripts initiate downstream of either consensus TATA box motifs or initiator elements, suggesting that host RNA polymerase II transcribes the viral mRNAs. The viral RNAs are polyadenylated and composed of multiple RNA species, indicating the complexity of geminiviral transcription [3].

The genomes of monopartite begomoviruses encode six genes, two in the virion-sense orientation (*V1* and *V2*) and four in the complementary-sense orientation (*C1* to *C4*). The *V1* gene encodes the coat protein (CP), involved in virus movement within and between plants [4,5], and the V2 protein which is involved in virus movement in plants as well as overcoming RNA silencing host defences triggered by dsRNA (also known as post-transcriptional gene silencing [PTGS]) for some virus species [6,7]. In the complementary-sense the *C1* gene encodes a rolling-circle replication initiator protein (known as the replication-associated protein [Rep]), that also interferes with host cell-cycle and is the only virus encoded protein required for virus replication, the C2 protein (known as the transcriptional-activator protein for some begomoviruses) that is involved in up-regulating late, virion-sense encoded genes (in some cases) as well as host genes and is a RNA silencing suppressor [6,8-10]. The product of the *C3* gene, known as the replication enhancer protein (REn), is involved in virus DNA replication (by interacting with Rep) and also interacts with host components [11]. The product of the *C4* gene may be a symptom determinant and also exhibits RNA silencing suppressor activity [6].

The betasatellites are a recently identified class of ssDNA satellites [2]. They are, in many cases, required by their helper begomoviruses to symptomatically infect the hosts from which they were isolated [12,13]. Betasatellites encode a dominant symptom/pathogenicity determinant (known as βC1) which is a suppressor of PTGS and may be involved in virus movement in plants and enhance virus DNA levels in plants [6,14-17].

Cotton leaf curl disease (CLCuD) is the most significant problem for cotton production across most of Pakistan and northwestern areas of India [18]. This disease appeared in epidemic form in 1991–92 and is caused by monopartite begomoviruses (seven distinct species were identified), often as multiple infections [19], and a specific betasatellite – Cotton leaf curl Multan betasatellite (CLCuMuB) [13]. In the late 1990’s the introduction of resistant cotton varieties restored cotton production in Pakistan to pre-epidemic levels. However, from 2001–2002 onwards, the disease appeared on all previously resistant varieties, an indication of the appearance of a strain of the disease with the ability to break resistance (now known as the “Burewala” strain) [20]. CLCuD in resistant cotton varieties has been shown to be associated with a single begomovirus, *Cotton leaf curl Burewala virus* (CLCuBuV), which has spread across most areas of Pakistan and into India [21-23]. CLCuBuV is a recombinant virus consisting of sequences derived from two parents, *Cotton leaf curl Kokhran virus* (CLCuKoV; which donated the virion-sense sequences) and *Cotton leaf curl Multan virus* (CLCuMuV; which donated the complementary-sense sequences). These two species were dominant in cotton prior to resistance breaking [19,21]. Significantly, CLCuBuV lacks an intact *C2* gene. This is surprising since all begomoviruses, curtoviruses and the only known topocurvirus reported to date have a *C2* gene that potentially encodes an ~134aa protein [21]. C2 is a multifunctional protein that plays an important role in host-virus interactions.

The betasatellite associated with CLCuBuV was also shown to be recombinant. This consists of the original CLCuMuB with a small fragment (~80 nt), in a non-coding sequence, derived from a betasatellite first identified in tomato [21,24]. In common with all betasatellites, CLCuMuB encodes a single gene, *βC1*.

The work presented here consisted of mapping the major transrcripts of CLCuBuV and its associated CLCuMuB for comparison to the transcript maps of other begomoviruses/betasatellites and investigating whether the recombination/mutation events involved in their evolution have affected gene expression at the level of transcription.

## Methods

### Infection of plants and RNA isolation

The begomovirus clones CLCuBuV–[PK:Veh2:4] (accession number AM421522) and associated betasatellite, CLCuMuB-[PK:Veh:06] (AM774307) were used to infect *N. benthamiana* plants as previously described [21].

Total RNA was isolated from plants using Trizol reagent (Gibco-BRL) as described by the manufacturer. The extracted RNA was dissolved in diethylpyrocarbonate-treated water and stored at −80°C.

### Northern blot hybridization

Total RNA (10 μg) was electrophoresed on 1.2% agarose MOPS gels, blotted onto nylon membranes (Hybond N+; Amersham) and UV cross-linked. DNA fragments for virus complementary-sense (coordinates 1059–66) and virion-sense (coordinates 124–1059) probes were PCR amplified using specific primers CF/CR and VF/VR (Table 1), respectively, and labeled with [α-^32^P]dCTP using a Megaprime labeling kit (Amersham). Hybridization was performed at 45°C for 16 h. Following stringent washing, radioactive signals were detected using a storage phosphor screen and the images were acquired after 3 h exposure using a Typhoon phosphoimager (Amersham). A betasatellite specific DNA probe was derived from the complete coding region of the *βC1* gene, labelled with a DIG PCR labelling kit and exposed to X-ray film after treatment with CDP-Star (Roche).

**Table 1:** Oligonucleotide primers used in the study

**Primer**	**Oligonucleotide sequence (5**^′^**to 3**^′^**)**	Nucleotide coordinates*
BuC2R	TCATTCAAGATCTACTCTCC	1500-1520
REn-^′^5B	GCTGTGAGGTCATCCAGATTC	1235-1256
V2CP-^′^5B	TAGGAACATCTGGACTTCTGT	488-466
V2cpB2	ACCGTGAACGGTGTCGGGGA	176-156
BC2	TGCTCCCTTCAAAGCCGT	358-376
BC2bb	GAGATCGAGATAGAAGATATAG	314-292
BC1-^′^3	CAAGTATGAAGGGATCGTCCA	435-415
BC1-^′^5	TGGACGATCCCTTCATACTTG	415-435
BuC2F	ATGCAACCTTCATCACTCTC	1608-1587
REn-^′^3B	ATGGATTCACGCACAGGGGA	1463-1443
5^′^RACE outer	GCTGATGGCGATGAATGAACACTG	-
5^′^RACE inner	CGCGGATCCGAACACTGCGTTTGCTGGCTTGATG	-
3^′^RACE outer	GCGAGCACAGAATTAATACGACT	-
3^′^RACE inner	CGCGGATCCGAATTAATACGACTCACTATAGG	-
C1C4-^′^5K	CTAGTTCCTTAATGACTC	2137-2155
C1C4-5^′^K2	AACGTTTGGGGGGAGCCAT	2577-2596
C1C4B1	ATGGCTCCCCCCAAACGTT	2596-2577
v2cpb1	ATGTCGAAGCGACCAGCAGATATAATC	292-318
c1c4b2	ATTGTCTCCAAATGGCAT	2664-2682
v2cpb2b	GCGTACCTTCGAAGCGGGCGTGGAAAT	320-346
v2cp-^′^3b	ACAGAAGTCCAGATGTTCCTAG	466-488
VF	CTTCGTTGCTAAGTTTGCG	105-123
VR	ATTTGTCACGGAATCATAGA	1059-1039
CF	GCGTCATATGATTGGCCGAC	66-46
CR	TATTGAAGATGATTGGTCTA	1076-1096

*CLCuBuV nucleotide coordinates are according to acc. no. AM421522 [21]. CLCuMuB nucleotide coordinates are according to acc. no. AM774307 [21].

### 5^′^ RNA ligase-mediated rapid amplification of cDNA ends (RLM-RACE) PCR

5^′^ RLM-RACE PCR was performed using an RLM-RACE Kit (Ambion) according to the manufacturer’s instructions. Total RNA was treated with alkaline phosphatase to remove the 5^′^ phosphate group of non-capped mRNAs, followed by treatment with tobacco acid pyrophosphatase (TAP) to decap mRNAs. TAP treated RNA was ligated to a synthetic 5^′^ RNA adapter (Table 1) using T4 RNA Ligase. This RNA was reverse transcribed and PCR was performed with specific primer combinations (Table 1) to amplify 5^′^ termini of virus and betasatellite specific transcripts.

### 3^′^ RLM-RACE PCR

3^′^ RLM-RACE PCR was performed according to the manufacturer’s instructions (Ambion). Total RNA (1 μg) was reverse transcribed with an oligo (dT) adapter primer (Table 1), and cDNA was used in PCR with specific primer combinations (Table 1) to amplify 3^′^ termini of virus and betasatellites specific transcripts.

### Cloning and sequencing of RLM-RACE PCR products

PCR products were purified using a gel extraction kit (Fermentas) according to the manufacturer’s instructions, and were cloned into the pTZ57R/T vector (Fermentas). PCR products (without cloning), as well as cloned products, were sequenced (Macrogen, Korea). Sequences were analyzed using the Lasergene software package (DNASTAR Inc.).

## Results

### Northern blot analysis of virus-specific RNAs in CLCuBuV/CLCuMuB-infected *N. benthamiana* plants

**Figure 1:**
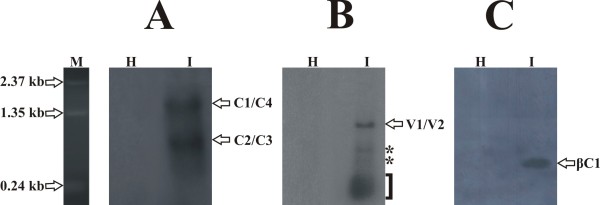
**Northern blot analysis of the transcripts of CLCuBuV.** Northern blot of total RNA extracted from a healthy *Nicotiana benthamiana* plant (H) and a plant infected with CLCuBuV/CLCuMuB (I). Detection of complementary- and virion-sense specific RNAs in infected plants are shown in panels A and B respectively. Immobilized RNA was hybridized to complementary- (**A**) and virion-sense probes (**B**). Detection of CLCuMuB specific RNA is shown in panel **C**. Approximately equal amounts (10μg) of RNA were loaded in each well. Estimated sizes are given in kilobases (kb) based on the positions of a co-electrophoresed RNA marker (M). Two additional hybridization signals (indicated by the asterisks) and the smear at the bottom of the virion-sense probed blot (indicated by the bracket on the right) is discussed in the text.

RNA gel blots of total RNA extracted from virus-infected *N. benthamiana* plants are shown in Figure 1. Hybridization with the CLCuBuV complementary-sense probe identified two major specific RNAs of ~1.7 and ~0.7 kb in CLCuBuV/CLCuMuB-infected plant tissue (Figure 1, panel A). Hybridization with the virion-sense probe revealed a single predominant RNA of ~1 kb in infected plants (Figure 1, panel B). No hybridization was detected in extracts from healthy, non-inoculated *N. benthamiana* tissue to either probe. In addition to the single long transcript on the blot probed with the virion-sense probe, a smear was observed at the bottom of the blots. This most likely represents degradation products of the major transcript (Figure 1, panel B). Two additional hybridization signals for virion-sense (of approximately 300 and 500nt) are shown by asterisks. The blots were repeated twice with independent samples and the same results were obtained each time.

### High-resolution mapping of the CLCuBuV complementary-sense transcripts

**Figure 2:**
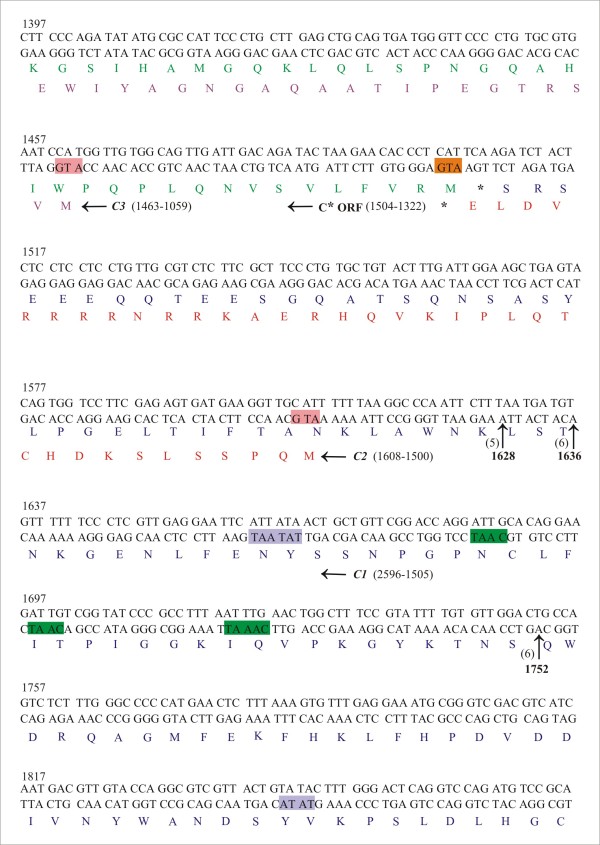
**Mapping the 5**^**′**^**ends of the short CLCuBuV *C2 *and *C3 *gene transcripts.** The nucleotide sequence of CLCuBuV between coordinates 1397–1876 is shown. The beginning of genes *C2* and *C3* are indicated by the start codon (ATG; highlighted in pink) and with the translations shown in red and purple text. The C-terminal end of the *C1* gene translation is shown in blue. The translation of a pseudo-gene (C*), resulting from the frame-shift mutation of the *C2* gene (in frame with the *C1* gene) is shown in green text with the start codon highlighted with an orange box. The putative promoter TATA elements and CAAT boxes are highlighted with purple and green boxes, respectively. The position of independent RACE clone ends mapping to the 5^′^ termini are indicated by their coordinates and with arrows. The numbers of cDNA clones sequenced for each end are indicated in brackets. Nucleotide numbering is according to [21].

**Figure 3:**
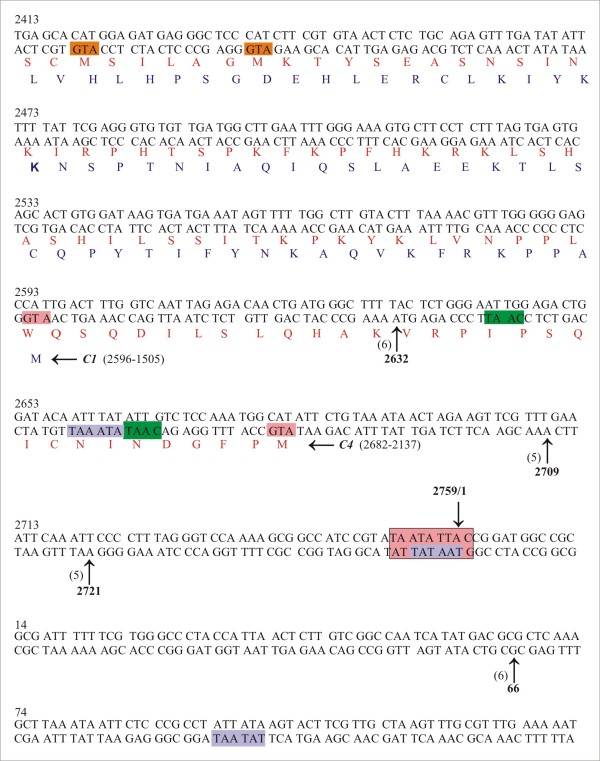
**Mapping the 5**^**′**^**ends of CLCuBuV *C1 *and *C4 *gene transcripts.** The nucleotide sequence of CLCuBuV between coordinates 2413–133 is shown. The beginning of genes *C1* and *C4* are indicated by the start codon (ATG; highlighted in pink). The predicted amino acid sequence of the C1 and C4 proteins are shown in blue and red text, respectively. The putative promoter TATA elements and CAAT boxes are highlighted with purple and green boxes, respectively. The position of independent RACE clone ends mapping to the 5^′^ termini are indicated with arrows and by their coordinates. The numbers of cDNA clones sequenced for each end are indicated in brackets. The conserved, between geminiviruses, nonanucleotide sequence (TAATATTAC) is highlighted with a large pink box. The two internal methionine codons (highlighted in orange) of the *C4* gene are discussed in the text. Nucleotide numbering is according to [21].

The location of 5^′^ ends of complementary-sense transcripts were determined by 5^′^ RLM-RACE PCR. Sequencing of clones generated by RLM-RACE, using the primer pair REn-5^′^B and 5^′^ RACE outer primer and then nested PCR using primer pair BuC2R and 5^′^ RACE inner primer (Table 1), indicated ligation of the RNA adapter primer to RNA with 5^′^ ends at positions 1628, 1636 and 1752. This transcript mapped upstream of the *C2* and *C3* and thus most likely represents the 5^′^ ends for the small CLCuBuV transcript of ~0.7-kb (Figure 2). Four other transcripts were identified, the 5^′^ ends of which mapped at positions 2632, 2709, 2721 and 66 using gene specific primer C1C4-5^′^K and 5^′^RACE outer primer and then using inner primer C1C4-5^′^K2 or c1c4b2 with 5′RACE inner primer (Table 1) in a nested PCR. The transcript mapped at position 2632 initiates upstream of the *C1* gene, while the longer transcript mapped at positions 2709, 2721 and 66 lies upstream of the *C4* gene (Figure 3).

**Figure 4:**
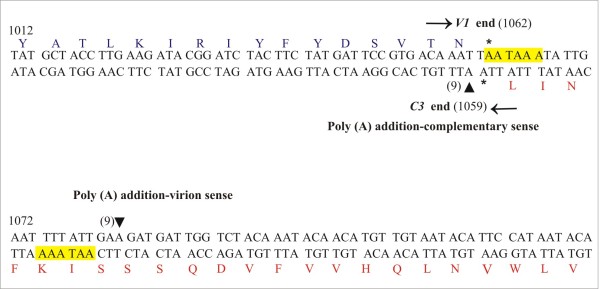
**Mapping the 3**^′^**ends of complementary- and virion-sense transcripts of CLCuBuV.** The nucleotide sequence of CLCuBuV between coordinates 1012–1131 is shown. The 3^′^ ends for transcripts are indicated by black triangles. The numbers of cDNA clones sequenced for each end are indicated in brackets. The translation stop codons of the *V1* and *C3* genes are indicated with asterisks. Polyadenylation signals are highlighted in yellow. Nucleotide numbering is according to [21].

The locations of 3^′^ ends for complementary-sense transcripts were examined by 3^′^RLM-RACE PCR. The gene specific primers C1C4B1 (outer) and BuC2F (inner) were used in combination with 5^′^ RACE outer and inner primers respectively for the 3^′^ end of ~1.7 kb transcripts. Similarly the outer primer BuC2F and inner primer REn-^′^3B were used for the 3^′^ end of small transcript of ~0.7 kb. Sequencing results showed that all the complementary-sense transcripts have a single major transcription termination site mapping at coordinate 1059 (Figure 4).

### High-resolution mapping of the CLCuBuV virion-sense transcripts

**Figure 5:**
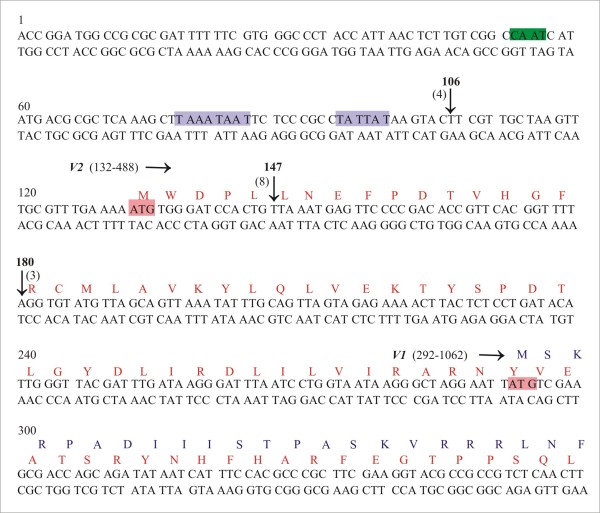
**Mapping the 5**^**′**^**ends of CLCuBuV *V1 *and *V2 *gene transcripts.** The nucleotide sequence of CLCuBuV between coordinates 1–360 is shown. The beginning of genes *V2* and *V1* are indicated by the start codon (ATG; highlighted in pink). The predicted amino acid sequence of the V1 and V2 proteins are shown in red and blue text, respectively. The putative promoter TATA elements and CAAT boxes are highlighted with purple and green boxes, respectively. The position of independent RACE clone ends mapping to the 5^′^ termini are indicated by their coordinates and with arrows. The numbers of cDNA clones sequenced for each end are indicated in brackets. Nucleotide numbering is according to [21].

The gene specific primer V2CP-^′^5B and the 5^′^RACE outer primer were used in the primary PCR and then primer v2cpb2b was used with the 5^′^RACE inner primer in a nested PCR to determine the 5^′^ends of virion-sense transcripts. Sequence analysis revealed the presence of two predominant 5^′^ ends, at nucleotides 106 and 147, for the virion-sense transcripts of CLCuBuV (Figure 5), which are consistent with the ~1 kb transcript determined by northern blotting (Figure 1). In addition a minor virion-sense transcript, mapping at nucleotide 180, was detected.

The 3^′^ RACE outer primer was used in combination with the gene specific primer v2cpb1 in the primary PCR and then nested PCR was performed using the 3^′^RACE inner primer and gene specific primer v2cp-^′^3b to determine the 3^′^ ends of virion-sense transcripts. The transcripts spanning genes *V1* and *V2* had the same predominant 3^′^ end with the transcription termination site mapping at nt 1083 and 3^′^ untranslated regions (UTRs) of 21nt (Figure 4).

### Mapping the 3^′^ and 5^′^ ends of βC1 transcript of CLCuMuB

**Figure 6:**
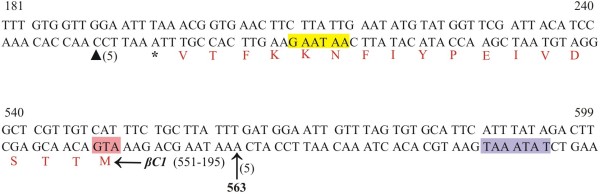
**Mapping the βC1 transcript of CLCuMuB.** The nucleotide sequence of CLCuMuB between coordinates 181–240 and 540–599 is shown. The beginning of the βC1 gene is indicated by the start codon (ATG; highlighted in pink) and the end by the stop codon (indicated with an asterisk). The numbers of cDNA clones sequenced for each end are indicated in brackets. The putative promoter TATA element is highlighted in purple and polyadenylation signal in yellow. The position of independent RACE clone ends mapping to the 5^′^ termini are indicated by arrows with coordinates and the 3^′^ termini by a black triangles. Nucleotide numbering is according to [21].

Sequence analysis of 5^′^ RLM-RACE clones for CLCuMuB revealed the presence of one predominant 5^′^ end. The primer pair BC2 and 5^′^ RACE outer primer were used in primary PCR and then nested PCR was performed using primer pair BC1-^′^5 and 5^′^ RACE inner primer (Table 1), indicated ligation of the RNA adapter primer to RNA with a 5^′^ end at position 563 (Figure 6).

3^′^ RLM-RACE clones showed transcription termination mapping at coordinate 190 (Figure 6). Gene specific primers BC1-3^′^ (outer) and BC2bb (inner) were used in combination with 3^′^ RACE outer and inner primers respectively (Table 1).

## Discussion

Although the transcription strategies of a few begomoviruses have been determined [25-29], including one monopartite begomovirus (*Tomato leaf curl virus* [ToLCV]) [28], none of the betasatellite-associated begomoviruses are amongst these. Only for two betasatellites, *Ageratum* yellow vein betasatellite (AYVB) and the non-recombinant CLCuMuB [15,30], have transcripts been determined. It was therefore of interest to determine the transcripts of CLCuBuV, and its associated recombinant betasatellite. Particular attention was paid to transcription across the mutation affecting the CLCuBuV *C2* gene to determine whether any transcriptional changes resulted from the mutation.

In common with other geminiviruses CLCuBuV produces multiple overlapping polycistronic RNA species that diverge from the IR, confirming a bidirectional transcription strategy. Transcription of the complementary-sense is more complex, producing multiple RNAs species with distinct 5^′^ ends and a common 3^′^ end. The polyadenylation sites are arranged such that complementary- and virion-sense RNAs overlap at their 3^′^ ends [29,31]. Though complementary and virion-sense transcripts overlap only by a small region, this unusual read-through transcription on a circular viral DNA could produce longer transcripts that are complementary, forming dsRNA, that may act to trigger RNA silencing [29,32]. Hybridization smears (Figure 1) detected on northern blots may represent the breakdown products of RNA formed from such aberrant read-through transcription. The additional hybridization signals detected on northern blots may represent short ORFs (Figure 1 panel, B). Such additional signals representing short ORFs have been mapped for the *BC1* transcription unit of *Mungbean yellow mosaic virus* (MYMV) [29]*.* These short ORFs may inhibit the translation of downstream genes unless leaky scanning occurs. Leaky scanning occurs when the 5^′^ most initiation codon has a weak context and ribosomes instead initiate translation at a downstream codon [33].

The virion-sense transcripts of CLCuBuV are comparable in size and location to those of other begomoviruses and the curtovirus *Spinach curly top virus* (SpCTV) [28,31]. Mapping of the virion-sense transcripts of CLCuBuV identified two major 5^′^ termini. One 5^′^ end mapped upstream of the *V1* and *V2* genes and could potentially direct the translation of both of these genes, whereas the second 5^′^ end mapped upstream of *V1*, and could potentially serve to translate only the CP (Figure 5). The transcript spanning the *V1*, with 5^′^ end mapped at coordinate 147, has an untranslated leader of 145 nt. An untranslated leader of ~160 nt has previously been reported for the *V1* gene of ACMV [25]. Analysis of the sequences of virion-sense transcripts of CLCuBuV indicated the presence of TATA box sequences at 34 and 49 nt upstream of the *V2* start methionine codon. The size of RNAs characterised here is consistent with the ~1 kb virion-sense RNA detected in infected tissue (Figure 1, panel B).

The analysis detected no RNAs that span only the *C3*, suggesting that REn is expressed only from polycistronic mRNAs. The transcripts spanning the *C2* and *C3* genes are of two classes, short (initiating at coordinates 1628 and 1636) and long (initiating at 1752)(Figure 2).The two transcripts have short 5^′^ UTRs of 20 and 28 nt, whereas the long transcript has a longer 5^′^ UTR of 144 nt (Figure 2). *Tomato golden mosaic virus* (TGMV) also transcribes to give two size classes of transcripts encompassing *C2* and *C3*[34]. For TGMV there are additional AUG initiation codons in the 5^′^ UTR of the longer *C2/C3* transcript. The first of these has the capacity to translate the C-terminal 122aa of the Rep, terminating after the *C2* initiation codon, was shown to be inhibitory for C2 and REn expression [34]. For CLCuBuV there is a single AUG initiation codon in the 5^′^ UTR of the 1752 transcript (Figure 2), out of frame with the *C1*, which terminates 11 aa before the *C2* AUG initiation codon. This is unlikely to significantly affect *C2* translation since the additional AUG initiation codon in the 5^′^ UTR is in an unfavourable context (tAtAAUGaaU) compared to the consensus context for dicot mRNAs (aaA[A/C]aAUGGCu) [35], whereas both the *C2* and *C3* AUG initiation codons are in a more favourable context (AAAAAAUGca and cAACcAUGGa). Thus in contrast to TGMV, CLCuBuV may translate both C2 and REn from both the size classes of *C2/C3* transcripts.

The mechanism by which REn is expressed is unclear. The gap between both the *C1* and (truncated) *C2* terminator codons and the start of *C3* suggests that it may involve reinitiation or internal initiation. For TGMV the translation of *C3* from the short transcript may occur by leaky scanning, since the *C3* initiation codon occurs before the *C2* terminator codon [34]. However, for CLCuBuV the frame-shift mutation has resulted in the *C2* termination codon occurring before the *C3* initiation codon and there is an ORF (indicated as C* in Figure 2; this ORF would not encode C2 amino acid sequences), in-frame with *C1* and immediately after the *C1* termination codon), which could possibly be expressed by leaky scanning through the *C2* initiation codon. Translation of the C* ORF might adversely affect *C3* expression. However, further studies will be required to investigate these possibilities.

It remains unclear why geminiviruses would express REn from multiple transcripts. A possible explanation is that this protein may be required in larger amounts than possible from a single transcript or its expression requires more subtle control. The protein may be required at several stages of the virus infection cycle and thus may be expressed at different times from different mRNAs.

The transcript 5^′^ ends identified represent heterogeneous initiation sites of a bicistronic mRNA encoding both *C2* and *C3*. The predicted sizes of RNAs initiating at the 5^′^ and 3^′^ends mapped here is consistent with the complementary-sense RNAs detected in infected tissue. Identified transcription start sites are located downstream of putative TATA boxes at optimal distances of 20 to 35 nt. These results suggest that CLCuBuV uses a bicistronic transcription strategy, in common with other geminiviruses, to translate C2 and REn from a single transcript [29]. This is supported by the fact that *C3* gene-specific primers did not reveal any major transcription start between the *C2* and *C3* start codons, or splicing to remove the upstream *C2* start codon.

**Figure 7:**
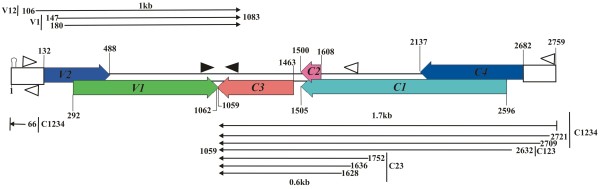
**Transcription map of CLCuBuV.** The diagram shows, in a linear form, the map positions of major virion and complementary sense transcripts (black arrows) relative to the intergenic region (open boxes), virion- and complementary sense genes (coloured arrows), TATA sequences (open triangles), and polyadenylation signals (shaded triangles). The start and stop coordinates of genes are indicated. Nucleotide numbering is according to [21].

The long (~1.7 kb) complementary-sense transcripts could potentially allow translation of the *C1*, truncated *C2* and *C3* genes (Figure 7). With the possible exception of the transcript initiating at coordinate 2632, all the long complementary-sense transcripts are also suitable for translation of *C4*. Transcript 2632 initiates downstream of the predicted AUG (coordinate 2682) of the *C4* gene and would thus appear not to direct translation of the C4 (Figures 3). However, CLCuBuV clone used here is unusual in encoding a predicted 181aa C4; most begomoviruses have a much smaller C4 (although the size is variable, typically ~85aa). Also, a minority of CLCuBuV isolates encode a predicted C4 of 100aa, although such clones have not so far been shown to be infectious to plants. The clone used here has two in-frame AUG codons (coordinates 2475 and 2493) within the *C4* sequence which might initiate the translation of products of 100 and 94aa, respectively, more in-keeping with the normal size of begomovirus C4 proteins. It is thus possible that the predicted long *C4* gene here is an artefact and that a more conventional C4 protein is translated from transcript 2632, or even that two distinct size classes of C4 protein are produced – 100aa from transcript 2632 and 181aa from the remaining transcripts. Which of these possibilities is correct will require further investigation.

For the long complementary-sense transcripts TATA and CAAT boxes are located at the requisite distances of approximately 30bp from the transcription initiation sites (Figure 3). The longer 1.7 kb transcript appears to use the invariant nonanucleotide sequence on the complementary strand as the promoter element (TATA box). Interestingly, the longest transcript maps at nt 66 with a 5^′^ UTR of 143 nt that spans the origin of replication and promoter elements far from the complementary-sense genes. A similar transcript has previously been observed for TGMV [36]. For CLCuBuV the short 5^′^ UTRs are 20–40 nt long and the promoter elements are located at an optimal distance from transcription initiation sites as reported previously for others geminiviruses and thus represent the authentic 5^′^ ends for these transcripts [31]. For the longer 5^′^ UTRs, these elements are far removed from the transcription initiation sites. It has been recognized that translation initiation efficiency from the start codon (AUG) depends on its position and sequence context within, and also on its distance from, the 5^′^ UTR [37]. Short 5^′^ UTRs may be associated with a decrease in translation initiation efficiency and vice versa [38].

The RACE clone sequences for the complementary-sense showed a single predominant 3^′^ end located between the converging *V1* and *C3* genes. Transcription termination occurs immediately following the stop codon for the *C3* gene. These transcripts do not have 3^′^ UTRs - polyadenylation occurs immediately following the stop codon of the *C3* gene. The poly (A) tail of the mRNA is added 21 nt downstream of canonical AATAAA polyadenylation signal. This site is located at an optimal distance downstream of a consensus polyadenylation termination signal suggesting that it represents the authentic 3^′^ end for these transcripts. Polyadenylation signals normally occur 10–30 nt upstream of the polyadenalytion site [15]. The 3^′^ UTR for virion-sense transcripts determined in this study are short, being 21 nt. The poly (A) tail is added 22 nt downstream of a polyadenylation signal. A similar arrangement has been observed for other geminiviruses; MYMV and SpCTV [29,31].

The single transcript identified for CLCuMuB maps with 5^′^ and 3^′^ ends at coordinates 563 and 190, respectively (Figure 6). These results are in agreement with the previous transcript mapping of the non-recombinant CLCuMuB [30]. The transcript is polyadenylated and the polyadenylation signal is 18 nt upstream of the stop codon. The putative polyadenylation signal of the non-recombinant CLCuMuB (AAATAA) differs from that of recombinant CLCuMuB (GAATAA). In contrast the putative TATA box element is the same for both CLCuMuB isolates, being 43 nt upstream of the start codon and 31 nt upstream of transcription start site. These results support the predicted TATA box and start codon for *βC1* and suggest that transcription may be initiated from a consensus TATA box sequence at an optimal distance of 20–35 nt. The 5^′^ UTR identified here is of 12 nt long and a transcript with a short leader sequence of eight nucleotides has previously been observed for AYVB [15]. The critical length of leader sequences is 7 nucleotides [39]. The differences between the results obtained here and those of previously obtained for AYVB [15] (multiple widely spaced 3^′^ termini and some minor 5^′^ termini) and for the non-recombinant CLCuMuB (one additional minor 3^′^ and 5^′^ terminus) [30], may be due to differences in experimental approach. For both previous analyses of *βC1* transcripts, RNA was extracted from transgenic plants harbouring dimeric betasatellite constructs – such plants would not be expected to contain episomally replicating satellite. The transcripts here are from a *bona fide* begomovirus-betasatellite infection. However, Saunders *et al*. [15] attributed some of the diversity in 3^′^ termini to the presence of a cryptic polyadenylation in the sequence of AYVB. No such cryptic polyadenylation signals (although the sequence of the presumed polyadenylation signal differs from the consensus, as mentioned earlier) are present in the recombinant CLCuMuB.

Sequencing of clones of the short transcript spanning the C2 mutation in CLCuBuV did not show any splicing, or other editing event, which might restore expression of a full-length C2 protein, supporting the conclusion of Amrao *et al*. [21] that an intact C2 is not expressed by CLCuBuV. However, the limited number of clones analyzed here and the limitations of the RLM-RACE technique, namely that it cannot produce the sequences of full length transcripts, means that the results must remain tentative. It may be desirable to further analyse CLCuBuV transcription, using for example circularization reverse transcriptase PCR, to determine the full length sequences of viral transcripts and identify any possible low abundance transcripts. Moreover, northern blotting and quantitative RT-PCR would also be helpful to compare the expression of early and late genes at various stages of replication cycle.

The transcription of CLCuBuV and the recombinant CLCuMuB is equivalent to earlier begomoviruses/betasatellites that were transcription mapped. The recombinations/mutations that led to their appearance caused no detectable differences at the transcription level. Nevertheless, the study provides some avenues of investigation to follow-up in the efforts to determine the mechanism of resistance breaking in cotton by CLCuBuV and its betasatellite. These include the possible differences in the C4 protein, or expression thereof, and possible effects on REn expression, in addition to the hypothesis put forward by Amrao *et al*. [21] that the C2 protein may have been the avirulence determinant of the pre-resistance breaking virus species that was recognized by resistant cotton. It is also important to note that the CLCuBuV REn is chimeric, consisting of sequences derived from both CLCuMuV and CLCuKoV [21]. These possibilities will be the subject of future investigation of the mechanism of resistance breaking in cotton.

## Competing interests

The authors declare that they have no competing interests.

## Authors’ contributions

FA performed the experiments and prepared the first draft of the manuscript. MS provided overall directions regarding the designing of all experiments, writing and supervised the work. FV supervised the hybridization experiments during the short-term visit of FA in Basel and discussed the different results with FA. RWB was involved in critical review of the work and in writing the manuscript. The final manuscript was read and approved by all authors.
